# Separate groups of dopamine neurons innervate caudate head and tail encoding flexible and stable value memories

**DOI:** 10.3389/fnana.2014.00120

**Published:** 2014-10-30

**Authors:** Hyoung F. Kim, Ali Ghazizadeh, Okihide Hikosaka

**Affiliations:** Laboratory of Sensorimotor Research, National Eye Institute – National Institutes of HealthBethesda, MD, USA

**Keywords:** nigrostriatal pathway, dopamine neuron, substantia nigra pars compacta, parallel circuit, object value learning, macaque monkey

## Abstract

Dopamine (DA) neurons are thought to be critical for reward value-based learning by modifying synaptic transmissions in the striatum. Yet, different regions of the striatum seem to guide different kinds of learning. Do DA neurons contribute to the regional differences of the striatum in learning? As a first step to answer this question, we examined whether the head and tail of the caudate nucleus of the monkey (*Macaca mulatta*) receive inputs from the same or different DA neurons. We chose these caudate regions because we previously showed that caudate head neurons learn values of visual objects quickly and flexibly, whereas caudate tail neurons learn object values slowly but retain them stably. Here we confirmed the functional difference by recording single neuronal activity while the monkey performed the flexible and stable value tasks, and then injected retrograde tracers in the functional domains of caudate head and tail. The projecting dopaminergic neurons were identified using tyrosine hydroxylase immunohistochemistry. We found that two groups of DA neurons in the substantia nigra pars compacta project largely separately to the caudate head and tail. These groups of DA neurons were mostly separated topographically: head-projecting neurons were located in the rostral-ventral-medial region, while tail-projecting neurons were located in the caudal-dorsal-lateral regions of the substantia nigra. Furthermore, they showed different morphological features: tail-projecting neurons were larger and less circular than head-projecting neurons. Our data raise the possibility that different groups of DA neurons selectively guide learning of flexible (short-term) and stable (long-term) memories of object values.

## INTRODUCTION

The basal ganglia are thought to be essential for the selection of action (Mink, 1996). Particularly relevant to this function is the striatum whose outputs could be used to facilitate a desired action (through the direct pathway) and inhibit undesired actions (through the indirect pathway) (Hikosaka et al., 2000; Frank et al., 2004; McHaffie et al., 2005). Notably, the striatum is a large structure which receives cognitive/sensorimotor/emotional signals from cortical areas (including association, sensorimotor, and limbic cortices) and subcortical areas [including thalamus and substantia nigra (SN)] (Selemon and Goldman-Rakic, 1985; Saint-Cyr et al., 1990; Parent and Hazrati, 1995; McHaffie et al., 2005). Hence, the basal ganglia are composed of multiple circuits for different kinds of action selection (Alexander et al., 1986). Such multiple mechanisms are likely to be deployed during learning of actions leading to rewarding outcomes. It has been suggested that two different regions of the striatum contribute to action learning differently (Hikosaka et al., 1999): the association striatum mainly in the early stage of learning; the sensorimotor striatum mainly in the late stage of learning. The association and sensorimotor striatum may correspond to the dorsomedial and dorsolateral striatum in rodents (Yin and Knowlton, 2006), and the rostral caudate/putamen and caudal putamen in monkeys (Miyachi et al., 1997, 2002) and humans (Lehéricy et al., 2005; Seger et al., 2010).

Our recent studies have shown that the caudate nucleus (CD) of the monkey also contains different learning mechanisms for choosing good objects. Neurons in the head of the caudate nucleus (CDh) change their responses to visual objects quickly depending on the reward values recently associated with the objects (flexible values), whereas neurons in the tail of the caudate nucleus (CDt) change their responses slowly depending on the reward values consistently associated with the objects (stable values; Kim and Hikosaka, 2013; Yamamoto et al., 2013). Both the flexible and stable value signals are sent to the superior colliculus via the SN pars reticulata (Hikosaka et al., 2014) and used to orient the monkey’s gaze to good (high-valued) objects (Kim and Hikosaka, 2013).

How does the object value learning occur in CDh and CDt? A key factor may be the input from dopamine (DA) neurons which carry reward-related signals (Schultz, 1998), heavily innervate the entire striatum (Richfield et al., 1987; Kato et al., 1995), and regulate synaptic plasticity in striatal neurons (Reynolds and Wickens, 2002). However, it is unknown how the object value learning occurs at different speeds in CDh and CDt. We considered two possible mechanisms. First, the difference may be caused by different learning mechanisms between CDh and CDt neurons, while they receive DA inputs non-selectively. Second, the difference may be caused by selective inputs from DA neurons carrying different learning signals. As a first step to test these hypotheses, we examined anatomically whether the fast-learning CDh and the slow-learning CDt receive inputs from the same or different DA neurons. Our data indicate that two spatially separate groups of DA neurons innervate CDh and CDt. Furthermore, these groups of DA neurons have different morphologies.

## MATERIALS AND METHODS

### GENERAL PROCEDURES

Three adult male rhesus monkeys (*Macaca mulatta*, 8–10 kg; monkey SM and ZO for neuronal recording and tracer study, and DW for neuronal recording) were used for the experiments. Monkeys DW and ZO were used in our previous study (Kim and Hikosaka, 2013), but we added more neuronal data in this study. All animal care and experimental procedures were approved by the National Eye Institute Animal Care and Use Committee and complied with the Public Health Service Policy on the humane care and use of laboratory animals. We implanted a plastic head holder and a recording chamber to the skull under general anesthesia and sterile surgical conditions. The chamber was tilted laterally by 25° and was aimed at CDh and CDt. Two search coils were surgically implanted under the conjunctiva of the eyes to record eye movements. After the monkeys fully recovered from surgery, we started training them with short-term flexible and long-term stable value procedures.

### NEURONAL RECORDING

While the monkey was performing a task, we recorded the activity of single neurons in different subregions of CD using conventional methods. The recording sites were determined with 1 mm spacing grid system, with the aid of MR images (4.7T, Bruker) obtained along the direction of the recording chamber. Single-unit recording was performed using glass-coated electrodes (Alpha–Omega). The electrode was inserted into the brain through a stainless-steel guide tube and advanced by an oil-driven micromanipulator (MO-97A, Narishige). The electric signal from the electrode was amplified with a band-pass filter (200–10 kHz; BAK). Neural spikes were isolated online using a custom voltage-time window discrimination software (MEX, Laboratory of Sensorimotor Research, National Eye Institute – National Institutes of Health [LSR/NEI/NIH]) and their timings were detected at 1 kHz. The waveforms of individual spikes were collected at 50 kHz.

### BEHAVIORAL TASKS

Behavioral tasks were controlled by a QNX-based real-time experimentation data acquisition system (REX, LSR/NEI/NIH). The monkey sat in a primate chair, facing a frontoparallel screen in a sound-attenuated and electrically shielded room. Visual stimuli generated by an active matrix liquid crystal display projector (PJ550, ViewSonic) were rear projected on the screen. We created the visual stimuli using fractal geometry. Their sizes were ∼8° × 8°.

### SHORT-TERM FLEXIBLE AND LONG-TERM STABLE BEHAVIORAL PROCEDURES

To find functional domains in CD, we used two behavioral procedures as previously described (Kim and Hikosaka, 2013).

#### Short-term flexible value procedure

This procedure allowed us to examine neuronal encoding of short-term flexible object values as they were being updated in blocks of trials. Therefore, learning (of object values) and testing (of the neuronal activity) were done in one task procedure. For each monkey, a fixed set of two fractal objects (say, A and B) was used as the saccade target. Each trial started with a central white dot presentation, which the monkey was required to fixate. After 700 ms, while the monkey was fixating on the central spot, A or B was chosen pseudo-randomly and was presented at the neuron’s preferred position or at the diagonally symmetric position. Before this procedure, we determined the neuron’s preferred position using a saccade task in which another fractal, as the target, was presented at multiple positions. During the flexible value procedure, the fixation spot disappeared 400 ms later, and then the monkey was required to make a saccade to the object within 4 s. The monkey received a liquid reward 300 ms after making a saccade to one object (e.g., A) but received no reward after making a saccade to the other object (e.g., B). During a block of 30–40 trials, the object–reward contingency was fixed, but it was reversed in a following block (e.g., B-high/A-low) without any external cue.

#### Long-term stable value procedure

To examine neuronal encoding of long-term stable object values, we conducted the learning procedure and the testing procedure separately on different days. In the learning procedure, the monkey experienced visual objects repeatedly in association with consistent reward values and thus learned their stable values. In the testing procedure, monkey’s saccade behavior and neuronal activity were examined using different tasks. To focus on stable object values, we applied the testing procedure to objects that had been learned for more than four daily sessions. Below we explain in detail (1) the learning procedure, and (2) the procedure for testing neuronal activity.

***Procedure for learning long-term stable object values.*** To create a fixed bias among fractal objects in their reward values, we used an object-directed saccade task. In each session, a set of eight fractals was used as the target and was presented at one of five positions (right, up, left, bottom, and center). The monkey made a saccade to the target to obtain a liquid reward. Half of the fractals were always associated with a liquid reward (high-valued objects), whereas the other half were associated with no reward (low-valued objects). One training session consisted of 160 trials (20 trials for each object). Each set was learned in one learning session in 1 day. The same sets of fractals were used repeatedly for learning across days (or months), throughout which each object remained to be either a high-valued object or a low-valued object. By the end of this study, monkeys SM, ZO, and DW learned the stable values of 272, 456, 608 objects, respectively.

***Procedure for testing neuronal activity.*** To test the neuronal coding of stable object values, we used a passive-viewing task. In each session, a set of eight fractal objects was used as the visual stimuli. While the monkey was fixating on a central spot of light, some of the fractal objects (*n* = 2–6) were chosen pseudo-randomly and presented sequentially in the neuron’s preferred position in a pseudorandom order (presentation time: 400 ms, inter-object interval time: 500–700 ms). The preferred position was determined using the passive-viewing task in which another fractal was presented at various positions. After every one to four object presentations, a reward was delivered 300 ms later. The reward was thus not associated with particular objects. Each object was presented at least six times in one session. The neuronal coding of stable object values was tested after long-term learning (more than four daily sessions) and after a sufficient retention period (>1 day after the last learning session). For each neuron, we used multiple sets of well-learned objects (2–4 sets, or 16–32 objects) to test its stable value-coding.

### NEURONAL VALUE-CODING

We analyzed the neuronal discriminations of high-valued and low-valued objects as previously described (Kim and Hikosaka, 2013). The statistical significance of the value-coding was determined for each neuron using ranksum test which compared the neuron’s responses (i.e., number of spikes during the object presentation) to high- and low-valued objects across trials. To illustrate the distribution of value-coding neurons in CD, we divided CD into 1 mm × 1 mm voxels (projected to the sagittal plane) and calculated the proportion of value-coding neurons (number of value-coding neurons divided by all object-responsive neurons) in each voxel.

To show the overall value-coding in CDh and CDt, we used the preferred and non-preferred responses to object values, since some CD neurons responded more strongly to high-valued objects (i.e., positive neurons) while others to low-valued objects (i.e., negative neurons). We first determined each neuron’s preferred value by comparing the magnitude of the neuron’s response to high-valued objects and to low-valued objects. This was done by computing a receiver operating characteristic (ROC) area based on the numbers of spikes within the test window in individual trials. We then averaged the responses of individual neurons in each subregion separately for the neurons’ preferred value and the non-preferred value. This was done by using a cross-validation method. Specifically, trials in one recording session were divided into the odd and even numbered trials. Either odd or even numbered trials were randomly chosen for determining the neuron’s preferred value (using the ROC analysis), and the other was used for computing the average response. The cross-validation method precluded any artificial result of neuronal discrimination due to an arbitrary choice of the preferred value.

### RETROGRADE TRACER INJECTION

To decide the injection sites, we recorded from single neurons throughout CD (except for CD genu) in monkey SM, ZO and DW, and examined if they encoded flexible or stable object values. Based on the value-coding map, we chose three injection sites in monkey SM and ZO: one in CDh and two in CDt. As retrograde tracers, we used Diamidino Yellow (DY; Sigma), Fast Blue (FB; Polyscience), and cholera toxin subunit B conjugated with Alexa 488 or Alexa 555 (CTB488 and CTB555; Life technologies). For FB, CTB488 and CTB555 injections, we used a custom-made injectrode consisting of an epoxy-coated tungsten microelectrode (FHC) for neuron recording and a silica tube (outer/inner tip diameter: 155/75 μm; Polymicro technologies) for tracer injection. For DY injection, we used a 30-gage stainless-steel needle. A 10-μL Hamilton syringe held in a manual infusion pump (Stoelting) was used to inject 0.3 μl FB (3% in distilled water), 0.3 μl CTB488, and 0.3 μl CTB555 (1% in 0.01 M, pH 7.4, phosphate buffer) at a speed of 0.01 μl/min and 1 μl or 0.6 μl DY (2% in 0.2 M, pH 7.2, phosphate buffer) at a speed of 0.02 μl/min. After the injection, the injectrode or injection needle was left for 1 h to minimize tracer diffusion along the injectrode/needle track. For monkey SM, each of DY, FB, and CTB555 were injected at two adjacent sites in CDh and CDt: 3 and 4 mm anterior to the anterior commissure (AC) in CDh (for DY); 8 and 9 mm (for FB) and 13 and 14 mm (for CTB555) posterior to AC in CDt. For monkey ZO, DY, CTB488, and CTB555 were injected at 3 mm anterior, 8 mm posterior, and 14 mm posterior to AC, respectively.

### HISTOLOGY

Two weeks after the tracer injection, monkey SM and ZO were deeply anesthetized with an overdose of sodium pentobarbital and perfused transcardially with saline followed by 4% paraformaldehyde. The head was fixed to the stereotaxic frame, and the brain was cut into blocks in the coronal plane including midbrain region. The block was post-fixed overnight at 4 C°, and then cryoprotected for 5 days in increasing gradients of glycerol solution (5, 10 to 20% glyceorol in PBS) before being frozen. Frozen block was cut every 50 μm using a microtome. Every 250 μm-interval slices were used for cell counting, and the adjacent two slices were used for Nissl and TH (tyrosine hydroxylase) staining. To examine the proportion of CTB- or DY-labeled neurons among all TH-positive neurons, we counted the TH-positive neurons in three representative slices in the rostral, middle, and caudal regions of SNc in each monkey and calculated the proportion.

### IMMUNOCYTOCHEMISTRY

To test if the CDt-projecting neurons were dopaminergic, we double-labeled 500 μm-interval SN slices with CTB and TH antibodies. After 1 h permeabilization with 0.05% Triton X-100 in TBS, the slices were blocked for 1 h in a solution containing 3% normal goat serum, 2% bovine serum albumin, and 0.05% Triton X-100 in TBS and then incubated with rabbit anti-CTB (1:1500; GenWay) and mouse anti-TH (1:3000; Immunostar) antibody overnight at room temperature. After three washes with PBS, the slices were incubated for 2 h at room temperature with goat anti-mouse IgG antibody conjugated with Alexa 488 for monkey SM or Alexa 647 for monkey ZO (1:200; Invitrogen) and goat anti-rabbit IgG conjugated with Alexa 594 for both monkeys (1:200; Invitrogen). The slices were air dried overnight at room temperature, and then mounted with VECTASHIELD (Vector). The cell images were scanned using fluorescence microscope (Zeiss AXIO imager M2). To test if the CDh-projecting neurons were dopaminergic, we examined co-localization of the TH signal and the remaining DY signal after the double labeling procedure.

### TOPOGRAPHICAL AND MORPHOLOGICAL ANALYSIS

A major goal of this analysis was to create two kinds of 3D reconstruction: (1) the injection sites together with the boundary of CD, (2) the locations of labeled neurons together with the boundary of SN. For each reconstruction we first plotted these locations in each coronal slice using a microscope digitizer system that has an encoder attached to the microscope stage (AccuStage). We then aligned the coronal slice images to the corresponding MR images and created a stack of 2D coronal slices. For 3D reconstruction of injection sites, we used IMOD, a 3D rendering program (Boulder Laboratory, University of Colorado, Boulder, CO, USA). For 3D reconstruction of labeled neurons in SNc, we used a Matlab program (MathWorks). To quantitatively analyze the topographical segregation of CDh-projecting neurons and CDt-projecting neurons, we used a discriminant analysis (Matlab, statistics toolbox).

A second goal was to analyze the morphology of CDh-projecting and CDt-projecting DA neurons. We plotted the cell soma of each CDh-projecting or CDt-projecting neuron based on TH immunoreactivity, and calculated its area and circularity using ImageJ (NIH), image processing and analysis program. We randomly sampled five projection neurons from each slice (500 μm interval) throughout SN of monkey ZO. The circularity was defined as follows: 4π × (cell area/perimeterˆ2). A circularity value of 1.0 indicates a perfect circle, whereas a smaller value indicates a more elongated shape.

## RESULTS

### DIFFERENCE IN OBJECT-VALUE LEARNING BETWEEN CDh AND CDt

**FIGURE 1:**
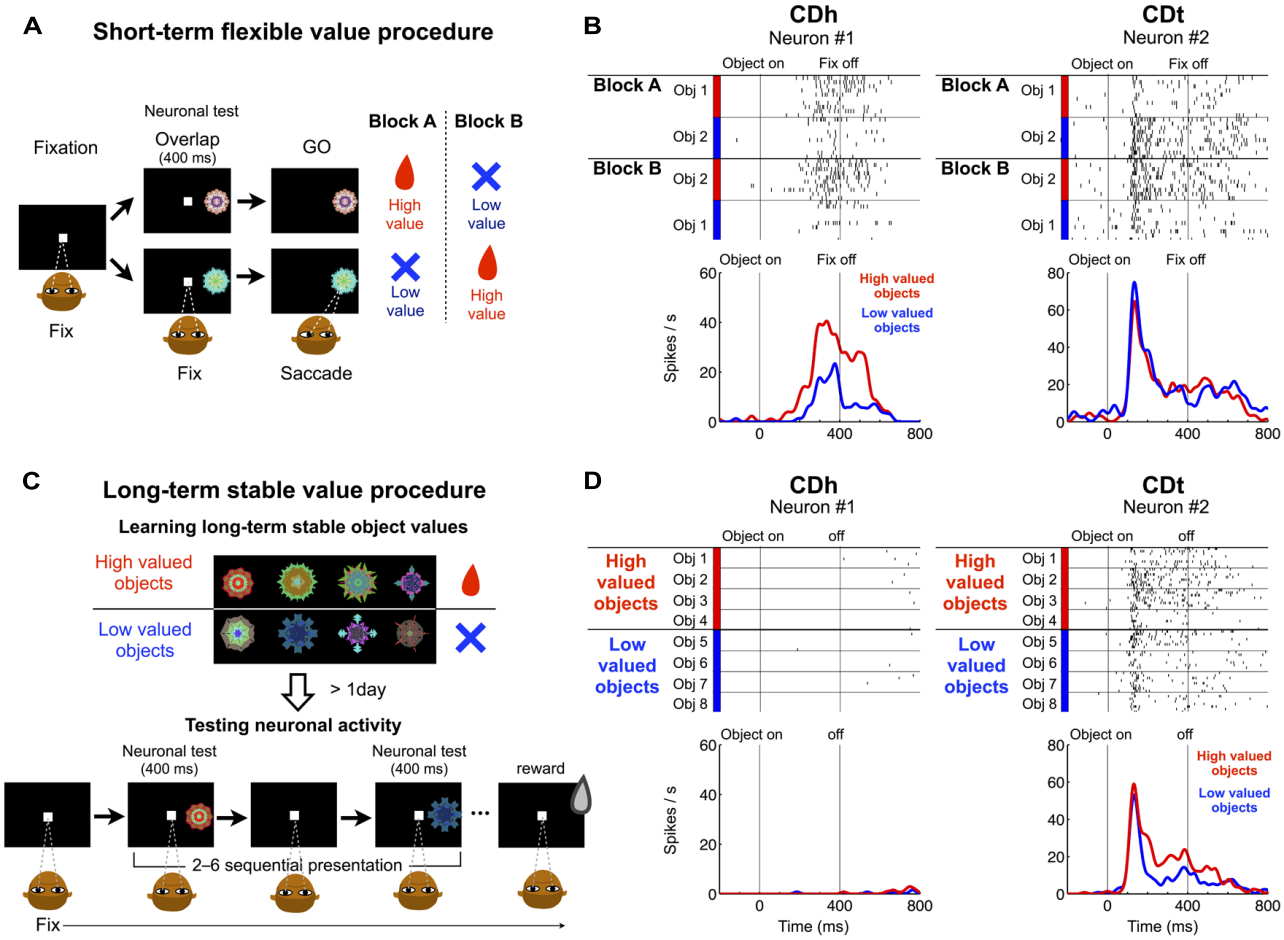
**Responses of CDh and CDt neurons to visual objects with flexible and stable values. (A)** In the short-term flexible value procedure, each of two fractal objects changed its value frequently across blocks of trials. On each trial one of two objects was presented, to which monkeys made a saccade, which was followed by a liquid reward or no reward. Neuronal recording was done simultaneously. **(B)** Reponses of one CDh neuron (left) and one CDt neuron (right) in the flexible value procedure. The CDh neuron responded each object more strongly when the object was flexibly high-valued (red) than low-valued (blue; *p* < 0.05, ranksum test). The CDt neuron responded to the two objects differently, but its response to each object was not affected by the flexible value changes. **(C)** In the long-term stable value procedure, each fractal object was associated with a high value (reward) or a low value (no reward) consistently during learning across days. The monkey experienced many sets of objects (eight fractals each), half high-valued and the other half low-valued (top). After the learning procedure, neuronal activity was tested on separate days using a fixation task in which the objects were presented one at a time with no contingent reward feedback (bottom). **(D)** Responses of the same CDh and CDt neurons as in **(B)**. The CDt neuron (right) responded more strongly to the stably high-valued objects (red) than stably low-valued objects (blue), although it showed some object selectivity (*p* < 0.05, ranksum test). The CDh neuron (left) showed little response to the stably valued objects.

**FIGURE 2:**
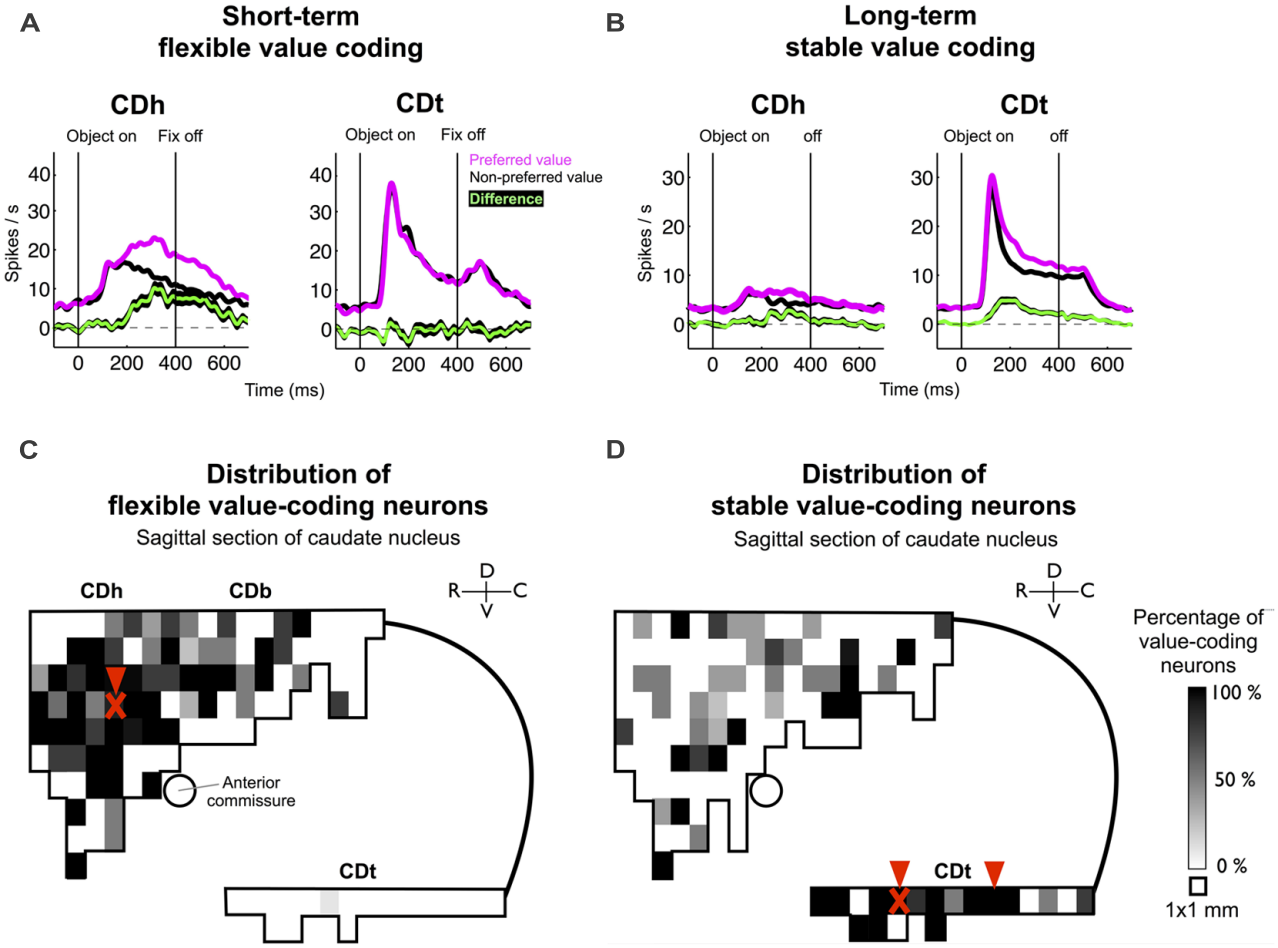
**Value-coding map of CD. (A)** Average responses to the flexibly valued objects of neurons in CDh (*n* = 71) and CDt (*n* = 54). Neuronal responses were averaged for the neurons’ preferred values (magenta) and non-preferred values (black) using a cross-validation method. The green line indicates the mean of the differences between the preferred and non-preferred responses, and the black line behind the green line indicates the standard error of the differences (mean ± SE). **(B)** Average responses to the stably valued objects of neurons in CDh (*n* = 32) and CDt (*n* = 59). Same format as in **(A)**. Data in **(A,B)** were obtained from monkey SM and ZO. **(C)** Value-coding map for short-term flexible values. **(D)** Value-coding map for long-term stable values. Data in **(C,D)** were obtained from monkey SM, ZO, and DW. A parasagittal plane across CD was divided into 1 mm × 1 mm areas, and the percentage of neurons that encoded each type of value significantly is shown for each area. Arrowheads indicate the injection sites of retrograde tracers. “X” marks indicate the locations of muscimol injections in monkey ZO which affected gaze orienting based on flexible and stable value learning (see Kim and Hikosaka, 2013).

Our main question was whether DA neurons contribute differentially to the processing of flexible and stable values of visual objects in CDh and CDt. As a first step to answer this question, we studied the origins of DA inputs by injecting retrograde tracers in the flexible value-coding and stable value-coding regions. To determine the injection sites, we examined (1) where visual object-responsive neurons were located in CD, and (2) whether these neurons encoded the values of the objects. In the first step, we confirmed our previous data: many neurons in CDh and CDt responded to visual stimuli (Hikosaka et al., 1989a; Yamamoto et al., 2012; Kim and Hikosaka, 2013). In CDt a majority of neurons were visually responsive; in CDh visually responsive neurons were often intermingled with non-visual neurons. Most CDt neurons responded to fractal objects differentially (i.e., object-selective); CDh neurons were less object-selective. In the second step, we examined whether these visually responsive neurons encoded flexible or stable values (**Figure 1**). The object values were created in two different ways (Kim and Hikosaka, 2013): (1) flexible values based on short-term object–reward association (**Figure 1A**), and (2) stable values based on long-term object–reward association (**Figure 1C**). **Figures 1B,D** show the responses of two example neurons, one in CDh and the other in CDt, to fractal objects in the flexible value procedure (**Figure 1B**) and the stable value procedure (**Figure 1D**). They responded to the objects differentially with respect to their flexible or stable values.

In the short-term flexible value procedure (**Figure 1A**), two objects changed their values (i.e., associated with reward or no reward) across blocks of trials. The CDh neuron responded more strongly to the object that had recently been associated with reward (**Figure 1B**-left). In other words, the CDh neuron’s response changed flexibly as an object changed its value. When two objects were presented simultaneously, the monkey made a saccade to the high-valued object in most trials to obtain the reward (Kim and Hikosaka, 2013). Thus, the CDh neuron’s response was correlated with the monkey’s choice preference among flexibly valued objects. In contrast, the CDt neuron responded to the two objects differently, but its responses were not affected by the flexible values of the objects (**Figure 1B**-right).

In the long-term stable value procedure, many objects retained their values stably during repeated learning across days (**Figure 1C**-top). To test the neuronal response, we presented the objects without reward feedback (**Figure 1C**-bottom). Thus, the values of these objects were solely based on long-term object-value learning. The CDt neuron overall responded more strongly to the high-valued objects than low-valued objects, although its response varied across objects (**Figure 1D**-right). When these objects were presented simultaneously, the monkey made saccades to high-valued objects even when no reward was given (Kim and Hikosaka, 2013). Thus, the CDt neuron’s response was correlated with the monkey’s choice preference among stably valued objects. In contrast, the CDh neuron showed no response to these objects, regardless of their stable values (**Figure 1D**-left).

### RETROGRADE TRACER INJECTIONS IN CDh-FLEXIBLE AND CDt-STABLE VALUE LEARNING SITES

The difference in object value-coding shown in **Figures 1B,D** was common among visually responsive neurons in CDh and CDt (**Figures 2A,B**), confirming our previous results (Kim and Hikosaka, 2013). However, CD contains many neurons that do not respond to visual stimuli but are sensitive to goal-directed behavior, especially in CDh (Hikosaka et al., 1989b). Therefore, to examine the role of DA neuron in object value learning, it was critical to determine the locations in each of CDh and CDt where the object value-coding neurons are most abundant. To answer this question, we explored the entire CD and identified neurons showing a statistically significant response bias with respect to the flexible or stable values. The distribution and density of such object value-coding neurons are shown in **Figure 2C** (flexible values) and **Figure 2D** (stable values).

The results indicate that the flexible value-coding neurons were relatively localized in the central region of CDh (**Figure 2C**). The stable value-coding neurons were commonly found in CDt, except for the most caudal portion (**Figure 2D**). These CDh and CDt sites were critical for gaze orienting behavior based on flexible and stable value learning: using one of the monkeys (monkey ZO), we previously showed that inactivation of CDh (red “X” mark in **Figure 2C**) induced a selective impairment in the flexible value-guided behavior (controlled behavior), whereas inactivation of CDt (red “X” mark in **Figure 2D**) induced a selective impairment in the stable value-guided behavior (controlled behavior; Kim and Hikosaka, 2013).

**FIGURE 3:**
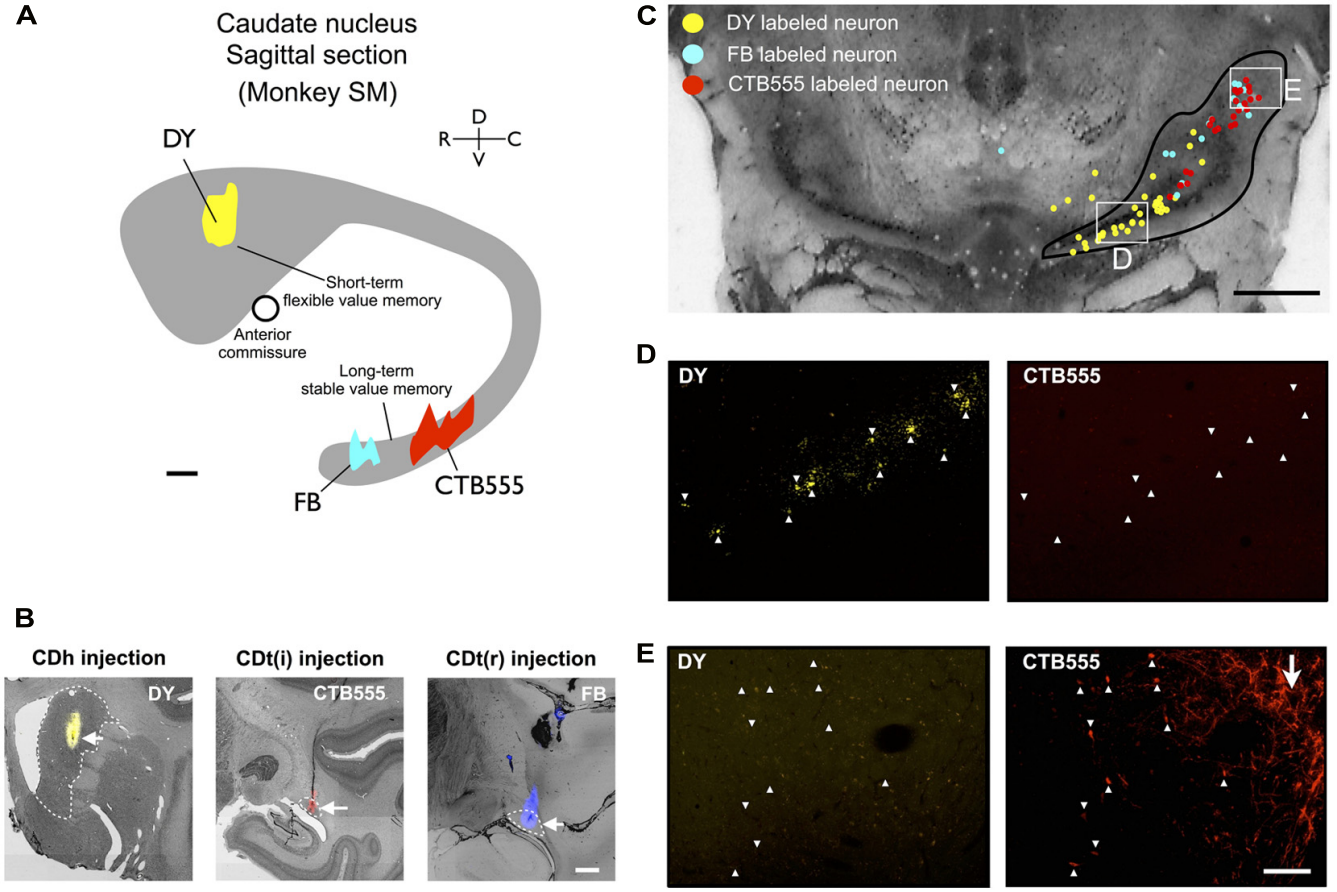
**Retrograde tracer injections in two functional domains of CD. (A)** Side view of CD showing injection sites of DY (in CDh), CTB555 and FB (in CDt) in monkey SM. Circle: anterior commissure. Scale bar: 2 mm. **(B)** Nissl-stained coronal sections showing the injection sites. White dotted lines indicate the borders of CDh and CDt. Scale bar: 2 mm. **(C)** Retrogradely labeled neurons in and around SN (Black line). Boxed regions are examined in **(D,E)**. Scale bar: 5 mm. **(D)** DY-labeled CDh-projecting neurons (left, indicated by white arrowheads) were not labeled with CTB555 (right). **(E)** CTB555-labeled CDt-projecting neurons (right) were not labeled with DY (left). White arrow indicates axon terminals of CDt neurons labeled with CTB555 anterogradely (right). Scale bar: 200 μm.

Based on the functional mapping, we injected retrograde tracers into the object value-coding sites in CDh and CDt (arrowheads in **Figures 2C,D**). Since the CDt site extends rostrocaudally, we injected tracers at two sites: rostral site [CDt(r)] and intermediate site [CDt(i)]. To examine the specific projections of DA neurons into CDh and CDt, different retrograde tracers were injected into the CDh and CDt (**Table 1**). **Figure 3** shows the injection sites and labeling patterns in monkey SM. Diamidino Yellow (DY) was injected into the flexible value-coding domain in the central region of CDh (**Figures 3A,B**-left). Cholera toxin B subunit conjugated with Alexa-555 dye (CTB555) and FB were injected into the CDt(i) and CDt(r) respectively (**Figures 3A,B**-middle and right).

**Table 1:** Retrograde tracers injected into CDh or CDt.

Monkey	CDh injection (Short-term flexible value-coding)	CDt(r) injection (Long-term stable value-coding)	CDt(i) injection (Long-term stable value-coding)
SM	Diamidino Yellow (DY)	Fast Blue (FB)	Cholera Toxin-Alexa555 (CTB555)
ZO	Diamidino Yellow (DY)	Cholera Toxin-Alexa488 (CTB488)	Cholera Toxin-Alexa555 (CTB555)

*Different kinds of retrograde tracers were injected into CDh, CDt(r) and CDt(i).*

### DISTRIBUTION OF CDh- AND CDt-PROJECTING NEURONS IN SNc

In the midbrain, retrogradely labeled neurons were found mainly in the ipsilateral substantia nigra pars compacta (SNc), but few in the ventral tegmental area (VTA) and contralateral SNc. Notably, neurons projecting to CDh and CDt were clustered in distinct regions of SNc. An example is shown in **Figure 3C**. CDh-projecting neurons were localized mainly in the ventral-medial region of SNc (yellow dots in **Figure 3C**), whereas CDt-projecting neurons were localized mainly in the dorsal-lateral region of SNc (cyan and red dots in **Figure 3C**). The CDh- and CDt-projecting areas (white boxes in **Figure 3C**) are enlarged in **Figures 3D,E**. The CDh-projecting area (ventral-medial SNc) contained DY-labeled cell nuclei (**Figure 3D**-left), but not CTB555-labled cell bodies (**Figure 3D**-right). The CDt-projecting area (dorsal-lateral SNc) contained CTB555-labled cell bodies (**Figure 3E**-right), but not DY-labeled cell nuclei (**Figure 3E**-left). These data suggested that separate groups of SNc neurons project to CDh and CDt.

Adjacent to the CDt-projecting area of SNc, we found a CTB55-labeled plexus which presumably represents anterogradely labeled axonal terminals of CDt neurons (**Figure 3E**-right). The plexus was located in the dorsal-lateral SNr. The result suggests that CDt neurons have direct output connections to dorsal-lateral SNr neurons and receive direct inputs from dorsal-lateral SNc neurons.

**FIGURE 4:**
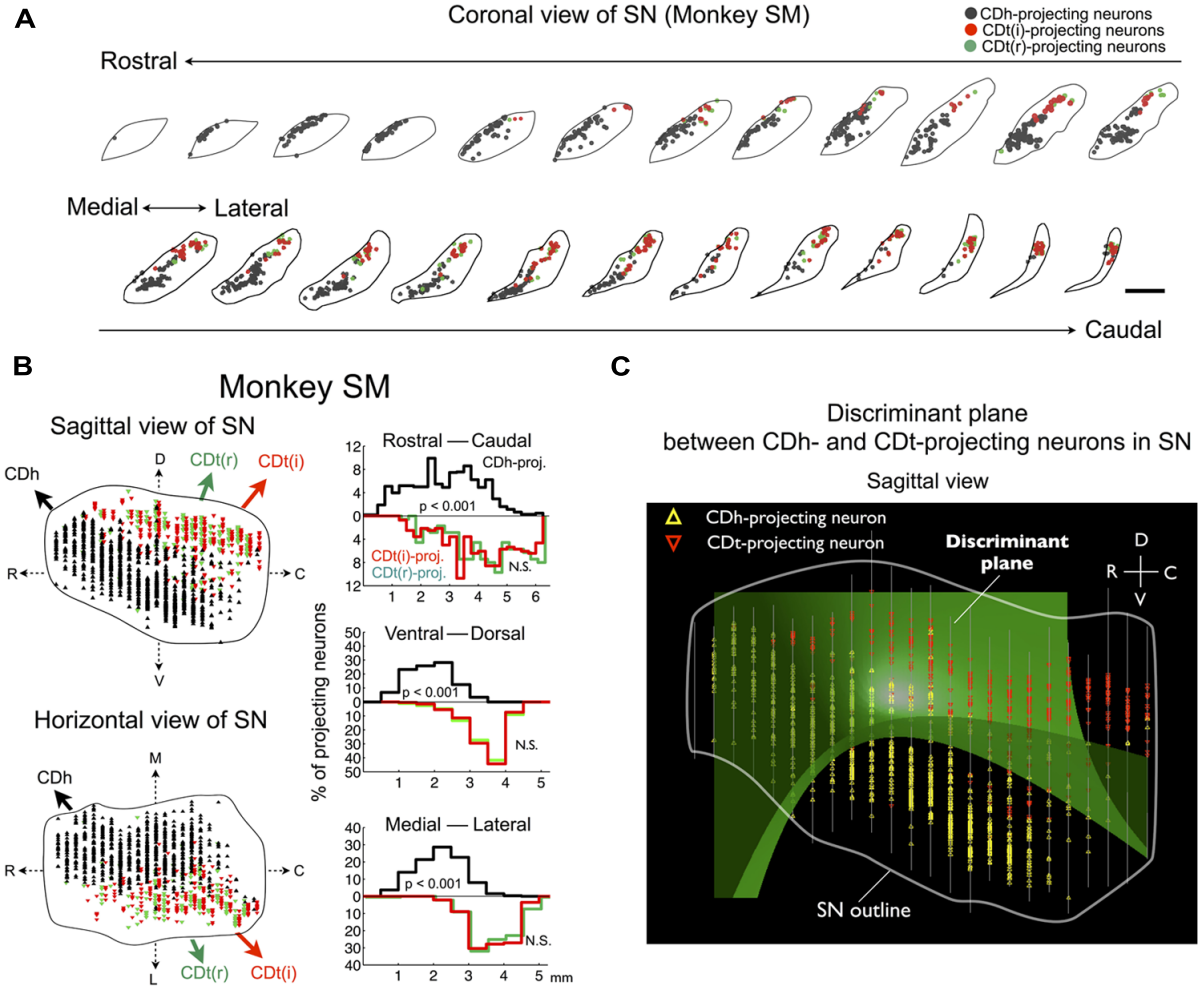
**Separate groups of SNc neurons project to CDh and CDt in monkey SM. (A)** CDh- and CDt-projecting neurons in SN. Coronal slices separated by 250 μm are shown from the rostral end to the caudal end of SN. Scale bar: 2 mm. **(B)** Distributions of CDh- and CDt-projecting neurons in sagittal (top) and horizontal (bottom) views (left). CDh-, CDt(i)-, and CDt(r)-projecting neurons are indicated by black, red, and green dots, respectively. Their distributions are projected to each of 3D axes (right). The P values were calculated by two-tailed *t*-test. **(C)** Discriminant analysis. CDh- and CDt-projecting neurons were best separated by a single quadratic plane (green). The neurons’ locations are projected to a parasagittal plane. White line indicates the border of SN. Between-slice interval: 250 μm

The dissociation of CDh- and CDt-projecting neurons was found in the entire SNc of monkey SM (**Figure 4A**). They were largely segregated in all of the three dimensional axes (**Figure 4B**-left): rostral-ventral-medial (r-v-m) SNc neurons projected to CDh, whereas caudal-dorsal-lateral (c-d-l) SNc neurons projected to CDt. The segregation of CDh- and CDt-projecting neurons in each axis of SN was statistically significant (*p* < 0.001, two-tailed *t*-test; **Figure 4B**-right). Discriminant analysis showed that CDh- and CDt-projecting neurons were largely separated into two groups by a single quadratic plane (green discriminant plane in **Figure 4C**; **Table 2**).

**Table 2:** Proportions of CDh and CDt-projecting neurons in two regions of SNc separated by a discriminant plane.

	CDh-projecting (%)	CDt-projecting (%)
**Monkey SM**
Group 1(c-d-l SNc)	8.4	88.9
Group 2(r-v-m SNc)	91.6	11.1
**Monkey ZO**
Group 1(c-d-l SNc)	10.2	83.0
Group 2(r-v-m SNc)	89.8	17.0

**FIGURE 5:**
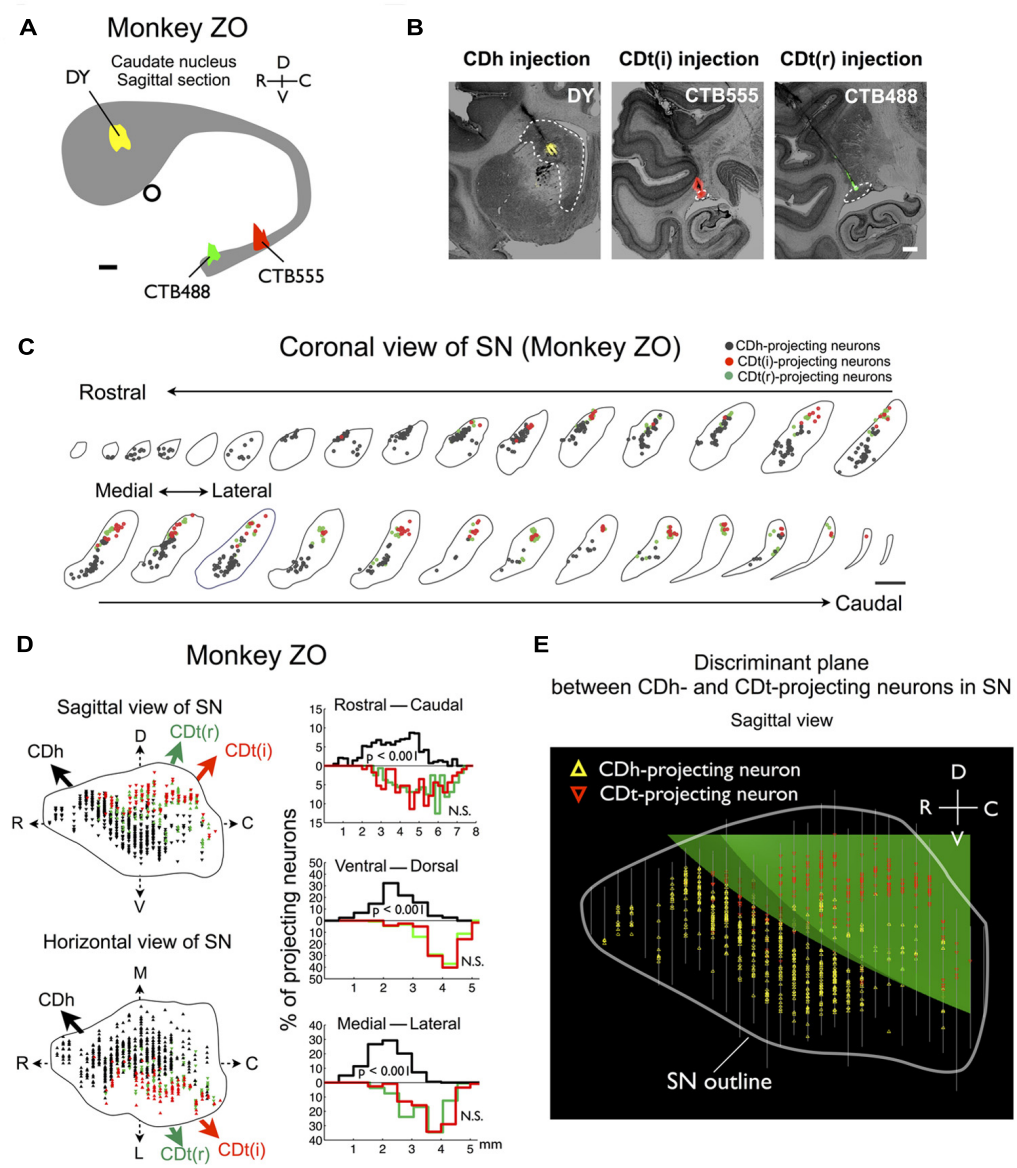
**Projections of SNc neurons to CDh and CDt in monkey ZO.** Same format as in **Figures 3** and **4**.

*Two groups of CDh- and CDt-projecting DA neurons are spatially separated by a discriminant analysis. Percentage indicates the proportion of CDh- and CDt-projecting neurons inside each discriminant plane (green plane in **Figures 4C and 5E**).*

We confirmed the same tendency in monkey ZO (**Figure 5**). DY was injected into the central part of CDh (yellow in **Figures 5A,B**). CTB555 and CTB488 (cholera toxin B subunit conjugated with Alexa-488 dye) were injected into CDt(i) and CDt(r) respectively (red and green in **Figures 5A,B**). CDh- and CDt-projecting neurons were separately localized in r-v-m SNc and c-d-l SNc, similarly to the distributions in monkey SM (**Figures 5C–E**). Notably, neurons projecting to both CDh and CDt were rarely found in monkey SM and ZO (**Table 3**). In addition, the CDt(r)- and CDt(i)-projecting neurons were mostly separated, although they were similarly localized in c-d-l SNc (**Table 4**; **Figures 4B and 5D**). These data indicated that separate groups of neurons in r-v-m SNc and c-d-l SNc innervated the flexible value-coding CDh and the stable value-coding CDt.

**Table 3:** Numbers of SNc neurons projecting to CDh, CDt, and both.

Monkey	CDh-projecting(# of cell)	CDt-projecting(# of cell)	Both(# of cell)	Both/CDh-projecting (%)	Both/CDt-projecting (%)
SM	1059	455	9	0.85	1.98
ZO	557	270	4	0.72	1.48

*Numbers indicate CDh-, CDt-, and both projecting neurons. Percentage indicates the proportion of both projecting neurons within CDh-projecting or CDt-projecting neurons.*

**Table 4:** Numbers of SNc neurons projecting to rostral CDt, intermediate CDt, and both.

Monkey	CDt(r)-projecting(# of cell)	CDt(i)-projecting(# of cell)	Both(# of cell)	Both/CDt(r)-projecting (%)	Both/CDt(i)-projecting (%)
SM	175	280	15	8.57	5.36
ZO	151	119	7	4.64	5.88

*Numbers indicate CDt(r)-, CDt(i)-, and both projecting neurons. Percentage indicates the proportion of both projecting neurons within CDt(r)-projecting or CDt(i)-projecting neurons.*

### MORPHOLOGICAL DIFFERENCE OF TH-POSITIVE DA NEURONS IN r-v-m AND c-d-l SNc

**FIGURE 6:**
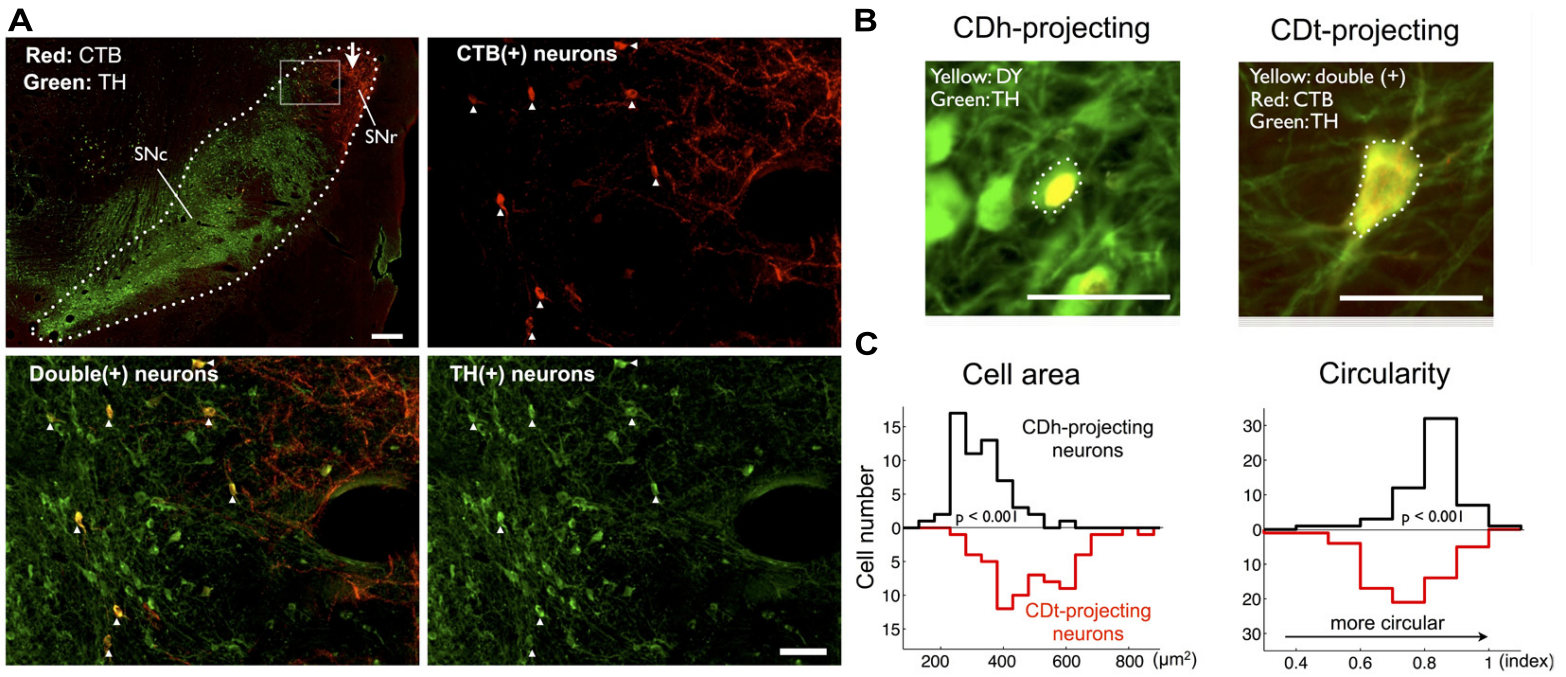
**CDt- and CDh-projecting neurons in SNc are dopaminergic. (A)** Distributions of CDt-projecting neurons (CTB-positive, red) and DA neurons (TH-positive, green) in monkey SM. The CDt-projecting neurons were clustered in the dorsolateral part of SN (dotted line in top-left section; scale bar: 500 μm), and this area (box in top-left) is enlarged in the other sections: CTB-positive (top-right), TH-positive (bottom-right), and merged (bottom-left; scale bar: 100 μm). The merged image (bottom-left) shows double-labeled neurons (yellow signal). White arrow indicates CTB-labeled axon terminals of CDt neurons (top-left). **(B)** Difference in cell area and circularity of CDh- and CDt-projecting DA neurons. Sample CDh- and CDt-projecting DA neurons (top) in monkey ZO. DY signal was localized in the nucleus (top-left), while CTB and TH signals were distributed in the whole cell (top-right). Dotted line indicates cell soma. Scale bar: 50 μm. **(C)** Left: CDt-projecting DA neurons had larger cell areas than CDh-projecting neurons (mean value: 467.6 vs. 304.2 μm^2^; *p* < 0.001, ranksum test; bottom-left). Right: CDh-projecting DA neurons were more circular than CDt-projecting neurons (mean value: 0.69 vs. 0.77; *p* < 0.001, ranksum test; bottom-right).

To test if the retrogradely labeled neurons are dopaminergic, we double-labeled SN sections (500 μm interval) with antibodies against CTB and DA-specific marker protein, TH (**Figure 6**). Anti-CTB antibody labeled the CDt-projecting neurons in SNc (**Figure 6A**, top-right, red dots). Anti-TH antibody labeled the cell soma and dendrites/axons of DA neurons (**Figure 6A**, bottom-right, green signals). Importantly, all the CTB-positive neurons (**Figure 6A**, top-right) were TH-positive (**Figure 6A**, bottom-right); they were double labeled (**Figure 6A**, bottom-left, yellow dots). This was true throughout the sections of SN, indicating that the CDt-projecting neurons were dopaminergic. The proportions of CDh- and CDt-projecting neurons among TH-positive neurons were 2.16 and 0.40% in monkey SM and 0.75 and 0.33% in monkey ZO. The CTB-labeled axon terminals of CDt neurons (**Figure 6A**, top-left and top-right, red plexus) were located in SNr and did not overlap with CDt-projecting DA neurons. CDh-projecting neurons were also likely to be dopaminergic because DY-positive neurons were TH-positive (**Figure 6B**-left). Notably, these CDh- and CDt-projecting DA neurons were different in morphological features. We compared the size and shape of cell bodies between DY and TH double positive neurons (**Figure 6B**-left) and CTB555-TH double positive neurons (**Figure 6B**-right): CDt-projecting DA neurons were larger but less circular than CDh-projecting DA neurons (**Figure 6C**).

## DISCUSSION

### TOPOGRAPHICAL ORGANIZATION OF DOPAMINE INNERVATION IN THE STRIATUM

Our retrograde tracer experiments suggested that largely separate groups of DA neurons in the monkey SNc (r-v-m SNc and c-d-l SNc) innervate CDh and CDt. It is possible that some of the retrograde labeling occurred via damaged axons en passage, especially because electrodes for neuronal recording were penetrated multiple times before the tracer injections. However, it is unlikely that such erroneous labeling heavily affected the results, because the retrograde labeling pattern inside SNc was similar between the two monkeys and the tracers were mainly placed inside CD.

Our result is consistent with the concept that DA innervation in the striatum is topographically organized: DA neurons in different regions of the SNc and VTA project to different regions of the striatum in monkeys and rodents (Haber et al., 2000; Joel and Weiner, 2000; Ikemoto, 2007). Notably, most anatomical studies have focused on the dorsal-ventral or medial-lateral difference in the striatum, and rarely examined the rostral–caudal difference. This is a missing link for DA functions, because, in monkeys and humans, the rostral–caudal regions of the striatum have been shown to participate in procedural learning at different stages (Hikosaka et al., 1999; Floyer-Lea and Matthews, 2004; Lehéricy et al., 2005; Tricomi et al., 2009; Seger et al., 2010) and action selection in different manners (Jankowski et al., 2009). In particular, the projections of DA neurons to subregions of CD, including CDt, have not been studied. Since CDt is prominent in primates (Hjornevik et al., 2007), a comparison of DA innervation between CDh and CDt may reveal a unique function of the primate DA system including humans.

Previous anatomical studies have shown that different groups of DA neurons innervate different regions of the striatum. However, it is unclear whether the differential DA innervation affects motor behavior differentially. Our results suggest that separate DA innervation controls a common motor output (i.e., gaze orienting) because CDh and CDt have downstream connections to SC through SNr (Hikosaka et al., 2000; Yamamoto et al., 2012; Yasuda et al., 2012). This suggests that a single motor action can be controlled differently by separate DA innervation. Indeed, our previous studies indicated that gaze orienting is influenced by the reward value of the target object in two different ways, which are processed in CDh and CDt separately (Kim and Hikosaka, 2013). Below, we will discuss this point further.

### DOPAMINE MECHANISMS UNDERLYING FAST AND SLOW-LEARNING

Our data showed fast-learning and forgetting of object values in CDh neurons (flexible value-coding) and slow-learning and forgetting in CDt neurons (stable value-coding; **Figures 1** and **2**; also see Kim and Hikosaka, 2013). Furthermore, the different time courses of learning in CDh and CDt might be caused by their separate inputs from the two groups of DA neurons in r-v-m SNc and c-d-l SNc. Below, we consider two hypothetical mechanisms.

A common concept of the DA mechanism is that the glutamatergic cortico-striatal synaptic transmission is enhanced or depressed by the repeated association or disassociation with DA inputs to the striatal neurons (Reynolds and Wickens, 2002; Surmeier et al., 2007). However, it is unclear how long the synaptic plasticity remains, especially during natural behavior. Our data, following the above concept, suggest that r-v-m SNc-DA neurons cause strong but short-lasting synaptic plasticity in the cortico-CDh transmission, whereas c-d-l SNc-DA neurons cause weak but long-lasting synaptic plasticity in the cortico-CDh transmission (mechanism #1). A critical question, then, would be: what determines the distinct time courses of the synaptic plasticity in CDh vs. CDt neurons. This remains to be addressed in future studies.

A simple assumption behind the above hypothesis is that both of the two groups of DA neurons send a common value signal, yet their outcomes are distinct (i.e., flexible vs. stable value-coding) due to the different time courses of synaptic plasticity. Alternatively, the two groups of DA neurons might send different kinds of signal to CDh and CDt, thereby causing the distinct value-codings (mechanism #2). A well characterized signal conveyed by DA neurons is reward prediction error (RPE) (Schultz, 1998), which represents an instantaneous change of reward values (i.e., actual reward value – expected reward value) and therefore is suited to generate flexible value-coding. Then, mechanism #2 predicts that r-v-m SNc-DA neurons encode RPE, but c-d-l SNc-DA neurons do not. The hypothetical difference in value-coding might be related to the morphological differences in cell soma (**Figures 6B,C**): CDt-projecting DA neurons in c-d-l SNc tended to be larger and less circular than CDh-projecting DA neurons in r-v-m SNc. Testing mechanism #2 requires further experiments, particularly recording activity of CDt-projecting DA neurons in c-d-l SNc.

### CIRCUIT MECHANISMS

The above hypothesis (mechanism #2) attempts to explain the difference in learning and memory between CDh and CDt by DA inputs, which basically reassigns the question to DA neurons. The real solution could instead be found in neuronal circuits. Our data showed that CDt neurons project their axons selectively to the caudal-dorsal-lateral portion of the SNr (c-d-l SNr) which was adjacent to the CDt-projecting DA neurons in c-d-l SNc (**Figure 6A**). It has been shown that GABAergic SNr neurons have axon collaterals to adjacent GABAergic SNr neurons as well as DAergic SNc neurons (Deniau et al., 1982; Tepper et al., 1995). These findings together suggest a loop circuit [CDt(GABA) → c-d-l SNr(GABA) → c-d-l SNc(DA) → CDt]. If this works as a positive loop, reward value signals might be retained through the loop circuit, providing a basis of long-term object-value memories.

A similar loop circuit may be formed for CDh-projecting DA neurons [i.e., CDh(GABA) → r-v-m SNr(GABA) → r-v-m SNc(DA) → CDh], because the distribution of CDh-projecting neurons (**Figures 4** and **5**) roughly matches the portion of SNr that receive inputs from CDh reported previously (Smith and Parent, 1986; Hikosaka et al., 1993). If this CDh loop circuit works similarly to CDt loop circuit (suggested above), it would also promote long-term object-value memories, but apparently it is not the case. This question remains an interesting issue for future research.

### IMPLICATIONS FOR DOPAMINE DISORDERS

Consistent with anatomical data described above, CDh-derived flexible value signals and CDt-derived stable value signals are mediated by different regions in SNr (c-d-l SNr and r-v-m SNr, respectively), both of which project to SC (Yasuda and Hikosaka, 2013). Experimental inactivation of the parallel circuits disrupted value-based gaze orienting, but in different manners (Kim and Hikosaka, 2013): CDh inactivation disrupted the preferred gaze orienting to objects that were immediately followed by a large reward (i.e., controlled orienting), whereas CDt inactivation disrupted the preferred gaze orienting to objects that had previously been associated with a large reward repeatedly but not currently (i.e., automatic orienting). This suggests the dichotomy of DA function: CDh-projecting DA neurons guide controlled behavior, whereas CDt-projecting DA neurons guide automatic behavior.

This suggestion provides an important clue to understanding DA dysfunctions. Patients with Parkinson’s disease may be impaired in cognitive and/or automatic behaviors (Brown and Marsden, 1990; Redgrave et al., 2010). Our study raises the possibility that impairments in cognitive behaviors may be caused by the degeneration of CDh-projecting DA neurons in r-v-m SNc, whereas impairments in automatic behaviors may be caused the degeneration of CDt-projecting DA neurons in c-d-l SNc.

In Parkinson’s disease, the degeneration of DA neurons tends to occur first in the lateral part of SNc (Rinne et al., 1989; Fearnley and Lees, 1991). We showed that larger and less circular DA neurons were localized in this lateral SNc (**Figure 6B**), suggesting that this type of DA neurons might be more vulnerable to cell death in Parkinson’s disease. Our data may provide an opportunity for differential diagnosis and treatments of Parkinson’s disease and other DA disorders.

## AUTHOR CONTRIBUTIONS

All authors, Hyoung F. Kim, Ali Ghazizadeh, and Okihide Hikosaka meet the four criteria for authorship.

## Conflict of Interest Statement

The authors declare that the research was conducted in the absence of any commercial or financial relationships that could be construed as a potential conflict of interest.
